# Supplementary material for: Diagnosis and Surgical Management for Advanced Pancreatic Cancer Requiring Vascular Resection
Source: Diagnostics (Basel). 2025 Dec 28;16(1):102. doi: 10.3390/diagnostics16010102 (PMC12785877; doi:10.3390/diagnostics16010102)
Supplement: Supplementary file 1 [file diagnostics-16-00102-s001.zip › diagnostics-4037187-supplementary.pdf]

Supplementary Material

Materials and Methods

The following search strings were used to identify relevant literature. Each string was applied in PubMed/MEDLINE and Scopus by two independent reviewers, with each reviewer responsible for searching one database. Final study inclusion was determined by consensus following discussion among all authors. The search was intended to be comprehensive but was not systematic, and the methods were not designed to adhere to PRISMA guidance.

Search string for diagnosing pancreatic adenocarcinoma (PDAC)

("Pancreatic Adenocarcinoma" OR PDAC OR "Pancreatic Cancer" OR Pancreatectomy) AND ("Borderline Resectable" OR BR OR "Locally Advanced" OR LA OR Resectable) AND (Diagnosis OR CT OR MRI OR Ultrasound OR "Endoscopic Ultrasound" OR EUS OR Diagnostic OR Imaging)

Search string for surgical management

("Pancreatic Adenocarcinoma" OR PDAC OR "Pancreatic Cancer" OR Pancreatectomy) AND ("Borderline Resectable" OR BR OR "Locally Advanced" OR LA OR Resectable) AND ("Surgical Management" OR "Vascular Resection" OR "Vascular Reconstructions" OR "Vein Resection" OR "Arterial Resection" OR "Venous Resection")

Search string for adjuvant/neoadjuvant therapy in PDAC management

("Pancreatic Adenocarcinoma" OR PDAC OR "Pancreatic Cancer" OR Pancreatectomy) AND ("Borderline Resectable" OR BR OR "Locally Advanced" OR LA OR Resectable) AND (Adjuvant OR "Adjuvant Chemotherapy" OR Neoadjuvant OR NAT OR "Neoadjuvant Chemotherapy" OR "Neoadjuvant Chemoradiotherapy" OR Chemoradiotherapy OR Radiotherapy)

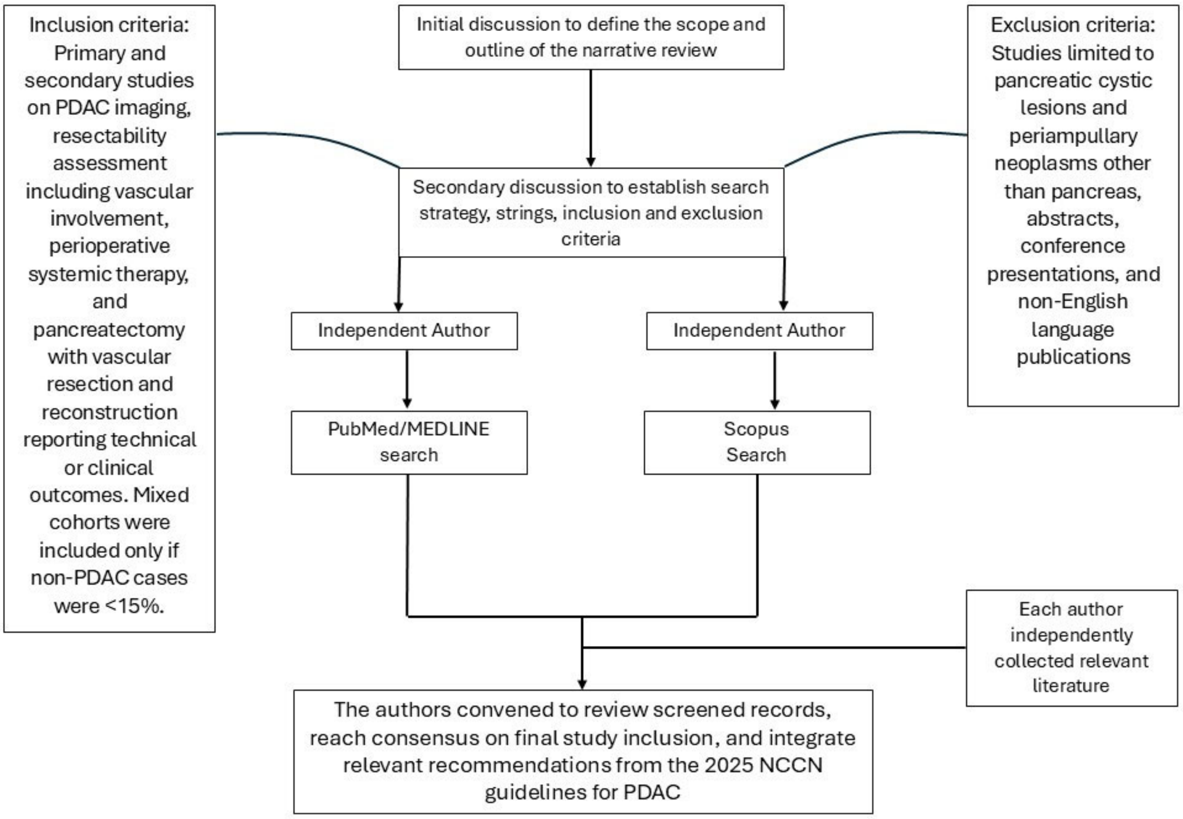

Supplementary Figure S1. Workflow for the identification and collection of relevant literature and data. The search was not performed systematically. PDAC = pancreatic ductal adenocarcinoma.

**Type of reconstruction by ISGPS**	**Definition—Reconstruction Technique**	**Relative Complexity and Complications Risk**
Type 1	Tangential excision with primary venorrhaphy.	Lowest
Type 2	Tangential excision with patch venoplasty.	Low–Moderate
Type 3	Segmental resection with primary end-to-end anastomosis.	Moderate–High

Type 4	Segmental resection with interposed conduit (interposition graft)	Highest

Supplementary Table S1. International Study Group for Pancreatic Surgery (ISGPS) venous resection (VR) and reconstruction classification. Complications risk refers to thrombotic events, occlusion/stenosis, blood loss.
